# The effect of amiloride in decreasing albuminuria in patients with diabetic kidney diseases: a prospective, crossover, open-label study

**DOI:** 10.1080/0886022X.2021.1892759

**Published:** 2021-03-03

**Authors:** Ruizhao Li, Zhiyong Xie, Li Zhang, Ying Huang, Jianchao Ma, Wei Dong, Zhilian Li, Yuanhan Chen, Huaban Liang, Yanhua Wu, Xingchen Zhao, Wenjian Wang, Zhiming Ye, Shuangxin Liu, Wei Shi, Xinling Liang

**Affiliations:** a Division of Nephrology, Guangdong Provincial People’s Hospital, Guangdong Academy of Medical Sciences, Guangzhou, China; b The Second School of Clinical Medicine, Southern Medical University, Guangzhou, China

**Keywords:** Diabetic kidney disease, amiloride, albuminuria, soluble urokinase-type plasminogen activator receptor

## Abstract

**Background:**

Diabetic kidney diseases (DKD) were the leading cause of End-stage renal diseases worldwide. Albuminuria was a target for treatment in DKD and decreasing albuminuria was particularly important for improving its prognosis. However, there is still a lack of specific treatment for DKD.

**Methods:**

We conducted a prospective, crossover, open-label study to investigate the effect of amiloride in patients with DKD. Safety and efficacy were assessed by monitoring urine protein creatinine ratio(uPCR), urinary albumin creatinine ratio (uACR), blood pressure, weight, serum sodium, serum potassium, cholesterol, triglyceride, uric acid, serum soluble urokinase-type plasminogen activator receptor (suPAR) and urinary suPAR. Ten subjects were enrolled in the trial.

**Results:**

In this prospective, crossover, open-label design, amiloride could induce a significant decrease of uACR in DKD. The decrease of serum and urinary suPAR in the amiloride/hydrochlorothiazide (HCTZ) group was also significant compared with those patients using HCTZ as the control group. Correlation analysis showed that the levels of urinary suPAR were positively associated with uPCR and uACR. No significant difference in blood pressure, weight, serum sodium, serum potassium, cholesterol, triglyceride, uric acid was seen between the amiloride/HCTZ group and the control group.

**Conclusion:**

In summary, among patients with DKD, amiloride could decrease albuminuria without severe side effects, which was accompanied by the significant decline of urinary suPAR.

## Introduction

Diabetic kidney diseases (DKD) were the leading cause of end-stage renal diseases and renal replacement therapy worldwide [1]. Recently the prevalence of DKD had been increasing dramatically in China and chronic kidney disease (CKD) related to diabetes had become more common than CKD related to glomerulonephritis [2]. The clinicopathological presentation of DKD was characterized by progressive albuminuria, hyperfiltration, associative glomerular injury, and tubulointerstitial fibrosis [3]. Moreover, persistent albuminuria could predict early deterioration of renal function [4] and was an independent risk factor of cardiovascular disease [5] and a target for the treatment of DKD [6]. Therefore, decrease albuminuria in DKD is particularly essential for improving its prognosis and reduce complications.

Albuminuria in DKD result from the damage of the glomerular filtration barrier, which leads to the excessive filtration of plasma protein and other macromolecular substances and exceeds the reabsorption ability of renal tubules. The podocyte plays a key role in the glomerular filtration barrier and its disruption results in a dramatic loss of function leading to albuminuria. The major biological functions of podocytes are to limit the passage of albumin from the circulation into the urine and to maintain overall glomerular integrity [7]. Thus, exploration of the drugs which target the improvement of the damaged podocyte would be conducive to improve albuminuria and delay the progression in DKD.

Recent studies have found that the increased expression of urokinase-type plasminogen activator receptor (uPAR) in podocytes could induce podocyte injury and albuminuria [8–12]. uPAR is required for activation of β3 integrin signaling within the kidney podocyte, which would lead to accelerated podocyte foot process dynamics and dysregulate the shape and function of the glomerular filtration barrier [11]. Increasing soluble urokinase-type plasminogen activator receptor (suPAR) levels in mice would induce podocyte abnormalities while reducing suPAR levels and blocking suPAR actions could improve kidney morphology [13].

Amiloride, a pyrazine compound inhibiting sodium reabsorption through sodium channels in renal epithelial cells, has been used as a diuretic in clinical practice. Our previous study had found that amiloride inhibited podocyte uPAR induction and reduced proteinuria in the 5/6 nephrectomy rat model and the LPS mouse model of transient proteinuria [14]. Moreover, amiloride could inhibit urine uPAR activity which attenuated plasminogen activation *in vivo* [15]. These results suggest that amiloride may have shown a potential effect on reducing albuminuria. Since there was no specific immunosuppressive therapy for DKD, we performed this prospective, crossover, open-label study to investigate the safety and effectiveness of amiloride in decreasing albuminuria in DKD.

## Materials and methods

### Subjects and data collection

Ten patients who were clinically or pathological diagnosed with DKD in Guangdong Provincial People’s Hospital from May 2018 to March 2019 and fulfilled the inclusion criteria were enrolled in this prospective, crossover, open-label study. DKD is clinical diagnosed in both type 1 and type 2 diabetes as the presence of persisting severely elevated albuminuria of >300 mg/24h, or a urine albumin-to-creatinine ratio (uACR) of >300 mg/gCr, with concurrent presence of diabetic retinopathy and absence of signs of other forms of renal disease. Moreover, DKD could also be pathologically diagnosed with morphological changes such as mesangial expansion and thickening of the glomerular and tubular basement membranes, as well as typical glomerulosclerosis with nodular mesangial lesions (Kimmelstiel–Wilson lesions). Among ten DKD patients enrolled, five patients were clinically diagnosed while five patients met pathological diagnostic criteria [16,17]. The inclusion criteria of this study were as follows: patients were over 14 years old; patients with good treatment compliance; urine protein creatinine ratio (uPCR) ≥500 mg/g Cr; patients signed written informed consent. Those who met the following criteria were excluded: eGFR ≤30 mL/min/1.73m^2^, previous intolerance or allergy to hydrochlorothiazide (HCTZ), patients with gout attack history within half a year, patients with active infectious diseases, patients with severe cardiopulmonary disease and central nervous system dysfunction, patients with a history of malignancy, patients’ life expectancy was less than 1 year, patients were pregnant, lactating or without contraceptive measures, patients who participated in other clinical trials within 3 months before enrollment, patients who were using immunosuppressive agents or glucocorticoids within 12 weeks before enrollment, patients failed to sign written informed consent or were unwilling to comply with the research protocol approved by the researcher. The procedures of this study were performed in accordance with medical ethics and the Helsinki Declaration and approved by the Ethics Committee of Guangdong Provincial People’s Hospital (No. GDREC2017318H). The registered clinical trial number was NCT03170336.

The clinical parameters were derived from electronic medical records and the hospital’s computerized database manually, including age, gender, weight, blood pressure, eGFR, serum creatine, uPCR, urinary albumin creatinine ratio (uACR), serum albumin, cholesterol, triglyceride, fasting glucose, uric acid, hemoglobin, platelet, serum sodium, serum potassium, serum suPAR, and urinary suPAR. The serum and urine specimens of the enrolled patients were collected before and after the treatment period and stored at −80° celsius. Serum suPAR and urinary suPAR were measured by the enzyme-linked immunosorbent assay (ELISA) method.

### Study design and outcome

**Figure 1.**
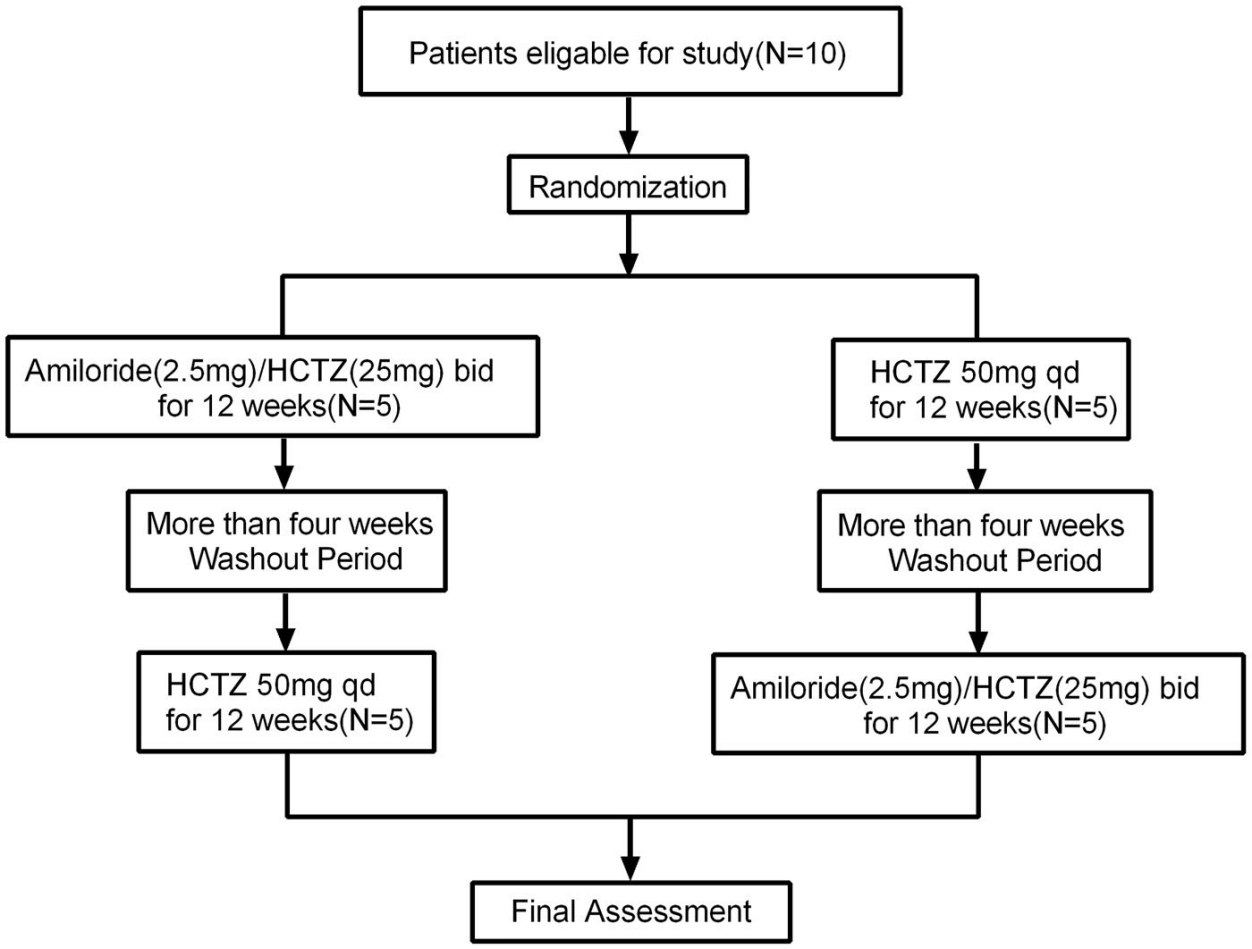
Flow chart of the patients enrolled in the study.

The study had a prospective, open-label, single-center, crossover design. In order to avoid hyperkalemia caused by amiloride, we used compound preparation of amiloride in this study, which contained 2.5 milligrams of amiloride and 25 milligrams of hydrochlorothiazide (HCTZ). Patients were orally administered with compound preparation of amiloride in doses of 5 mg of amiloride and 50 mg of HCTZ daily for 12 weeks treatment periods or with HCTZ in doses of 50 mg daily for 12 weeks treatment periods. Following more than 4-week washout periods, participants crossed over to the other treatment for 12 weeks. Outcomes were assessed at the end of each 12 weeks active treatment period (Figure 1). The randomization was performed centrally and patients were allocated to the different treatment sequences using random numbers. Guangdong Provincial People’s Hospital was responsible for coordinating and managing the investigational drug inventory, storage, distribution, and record-keeping for this clinical research study. This crossover study examined the amiloride’s effect in decreasing albuminuria in DKD patients as the primary outcome. The secondary outcome was the changes in blood pressure, weight, serum sodium, serum potassium, fasting glucose, serum uric acid, cholesterol, triglycerides before and after treatment.

### Detection of serum and urinary suPAR

Serum and urinary suPAR were detected using ELISA (JingMei, Hangzhou, China).

Blank wells and testing sample wells were set separately and 50 µL standard was added to the standard well. 40 μL sample dilution and 10 µL testing sample were added to the testing sample well. 100 µL HRP-Conjugate reagent were added to each well, except blank well. After closing the plate with the closure plate membrane, the plate was incubated for 60 min at 37 °C. The 20-fold wash solution was diluted into one-fold with distilled water and reserved. The closure plate membrane was uncovered and the liquid was discarded. Washing buffer was added to every well for 30 s and then were drained with 5 times repeat. 450 µL Chromogen Solution A and 50 µL Chromogen Solution B were added to each well, then evade the light preservation for 15 min at 37 °C. After that, 50 µL stop solutions were added to each well. The color change is measured spectrophotometrically at a wavelength of 450 nm. The concentration of suPAR in samples is then determined by comparing the OD of the samples to the standard curve.

### Statistical analyses

All data were analyzed using SPSS statistical software for Windows, version 23.0 (SPSS, Inc., Chicago, IL, USA). The measurement data accorded with normal distribution were expressed as mean ± SD and differences between the two groups were compared using Student’s *t*-test. The non-normally distributed data were expressed as medians (25th, 75th percentiles) and differences between two groups were compared using nonparametric Mann-Whitney U test. Categorical variables were compared using the *χ*^2^ test or *Fisher* exact test. Analysis of variance for two-period crossover design was performed to evaluate the therapeutic outcome and side effects. Correlation analysis was used to explore the correlation between variables. Two-tailed tests were used for all comparisons and a *p* < 0.05 was considered to be statistically significant.

## Results

### Comparison of the pretherapeutic baseline characteristics between patients received amiloride/HCTZ and HCTZ

Among ten DKD patients enrolled, five patients received amiloride/HCTZ, and after more than 4-week washout periods they were orally administrated with HCTZ. Another five patients were orally administrated with HCTZ and after more than 4-week washout periods they received amiloride/HCTZ. Mean age (±SD) was 57.1 ± 12.7 and 5 (50%) were men. Table 1 shows the participant baseline characteristics between two groups (Amiloride/HCTZ vs HCTZ) before treatment. There was no difference in pretherapeutic weight, blood pressure, eGFR, serum creatine, uPCR, uACR, serum albumin, cholesterol, triglyceride, fasting glucose, unic acid, hemoglobin, platelet, serum sodium, serum potassium, serum suPAR and urinary suPAR between patients received amiloride/HCTZ and HCTZ.

**Table 1.** Comparison of the baseline characteristics between patients received amiloride/HCTZ and HCTZ before treatment.

	All subjects (*n* = 20)	Amiloride/HCTZ (*n* = 10)	HCTZ (*n* = 10)	*p*-Value
Weight (kg)	64.7 ± 7.1	64.8 ± 7.3	64.7 ± 7.4	0.993
SBP (mmHg)	133.5 (125.0, 137.8)	135.0 (125.0, 137.0)	131.5 (123.5, 141.5)	0.912
DBP (mmHg)	74.5 (70.5, 77.8)	74.5 (70.0, 76.5)	74.0 (71.5, 78.5)	0.631
eGFR (mL/min/1.73m^2^)	52.8 ± 15.8	54.4 ± 13.0	51.2 ± 18.8	0.661
Serum creatine (μmol/L)	125.4 ± 47.8	116.3 ± 30.7	134.5 ± 60.7	0.412
uPCR (mg/g Cr)	3932.9 ± 2562.0	4607.8 ± 3017.4	3258.0 ± 1934.0	0.252
uACR (mg/g Cr)	2253.5 ± 1447.2	2678.3 ± 1737.8	1828.6 ± 1000.0	0.201
Serum albumin (g/L)	35.6 ± 4.6	34.6 ± 4.3	36.5 ± 4.8	0.355
Cholesterol (mmol/L)	5.2 ± 1.2	5.2 ± 1.4	5.1 ± 1.0	0.817
Triglyceride (mmol/L)	2.0 ± 1.1	1.9 ± 0.8	2.1 ± 1.4	0.624
Fasting glucose (mmol/L)	8.9 ± 4.1	8.7 ± 3.2	9.1 ± 5.0	0.822
Uric acid (mmol/L)	366.5 ± 111.5	352.1 ± 93.7	380.9 ± 130.4	0.578
Hemoglobin (g/L)	115.6 ± 21.4	114.6 ± 24.7	116.5 ± 18.7	0.849
Platelet (×10^9^/L)	241.4 ± 62.0	231.3 ± 52.8	251.4 ± 71.5	0.484
Serum sodium (mmol/L)	140.6 ± 3.1	140.8 ± 2.0	140.4 ± 4.0	0.813
Serum potassium (mmol/L)	4.5 ± 0.4	4.4 ± 0.3	4.6 ± 0.4	0.242
Serum suPAR (ng/mL)	155.6 ± 20.5	159.0 ± 19.4	152.2 ± 21.9	0.470
Urinary suPAR (ng/mmol)	49.2 ± 27.3	58.1 ± 32.7	40.2 ± 18.1	0.147

SBP: Systolic blood pressure; DBP: Diastolic blood pressure; eGFR: estimated glomerular filtration rate; uPCR: urine protein creatinine ratio; uACR: urinary albumin creatinine ratio; suPAR: soluble urokinase-type plasminogen activator receptor.

### Amiloride’s effect in decreasing uACR and suPAR

After the treatment period of 12 weeks, uACR, serum, and urinary suPAR decreased significantly in patients who received amiloride/HCTZ compared with those administrated with HCTZ. The decline in uACR over the 12 weeks after treatment was 605.0 (172.0, 1395.5) mg/gCr in the amiloride/HCTZ group and −353.0(−1090.3, 502.3) mg/gCr in the HCTZ group, with a resulting treatment difference between two groups (*F* = 8.279, *p* = 0.021). However, the decline in uPCR over the 12 weeks after treatment was 1385.5 (518.5, 3218.0) mg/gCr in the amiloride/HCTZ group and −390.0(−1402.5, 1185.8) mg/gCr in the HCTZ group, without a significant difference between groups (*F* = 5.131, *p* = 0.053). Moreover, the levels of serum suPAR decreased by 7.1 (−2.7, 14.9) ng/mL from the baseline while the decrease of urinary suPAR was 5.2 (1.8, 49.7) in the amiloride/HCTZ group, with a significant difference compared with the HCTZ groups (serum suPAR: *F* = 32.313, *p* < 0.001; urinary suPAR: *F* = 6.188, *p* = 0.038) (Table 2).

**Table 2.** Comparison of outcomes between amiloride and HCTZ over the course of the study.

	Amiloride/HCTZ(*N* = 10)	HCTZ(*N* = 10)	*F*-value	*p*-Value
uPCR (mg/gCr)			5.131	0.053
Start of treatment	4514.0 (1935.5, 7649.5)	2766.5 (1668.5, 4762.8)		
End of treatment	2030.5 (789.3, 5358.5)	3268.5 (2443.5, 4824.8)		
Changes of uPCR	−1385.5 (−3218.0, −518.5)	390.0 (−1185.8, 1402.5)		
uACR (mg/gCr)			8.279	0.021
Start of treatment	2571.0 (1163.5, 4525.8)	1558.0 (966.8, 2662.3)		
End of treatment	1327.0 (486.8, 3155.0)	2087.0 (1477.3, 2854.3)		
Changes of uACR	−605.0 (−1395.5, −172.0)	353.0 (−502.3, 1090.3)		
Serum suPAR (ng/mL)			32.313	<0.001
Start of treatment	149.7 (145.4, 176.4)	153.6 (134.4, 165.9)		
End of treatment	147.6 (137.4, 164.4)	152.1 (139.5, 178.4)		
Changes of serum suPAR	−7.1 (−14.9, 2.7)	4.0 (−6.4, 13.9)		
Urinary suPAR (ng/mmol)			6.188	0.038
Start of treatment	44.7 (34.9, 80.4)	37.9 (30.6, 50.7)		
End of treatment	31.2 (27.4, 36.7)	44.3 (20.3, 67.3)		
Changes of Urinary suPAR	−5.2 (−49.7, −1.8)	2.7 (−17.2, 28.1)		

uPCR: urine protein creatinine ratio; uACR: urinary albumin creatinine ratio; suPAR: soluble urokinase-type plasminogen activator receptor.

### Association between urinary suPAR and uPCR/uACR

**Figure 2.**
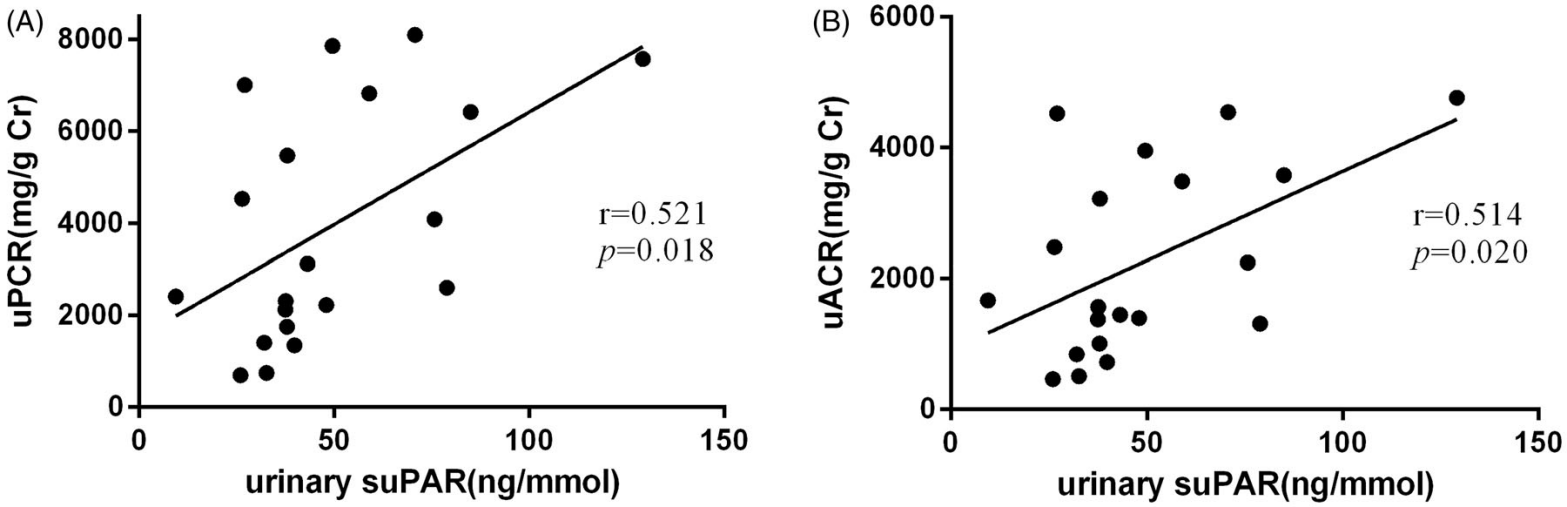
(A, B) The correlation between urinary protein/albumin creatinine ratio and urinary suPAR.

Correlation analysis showed that the baseline urinary suPAR was positively associated with uPCR (*r* = 0.521, *p* = 0.018) (Figure 2(A)). Additionally, the baseline urinary suPAR was also positively associated with uACR (*r* = 0.514, *p* = 0.020) (Figure 2(B)). However, neither uPCR nor uACR was found to have a significant association with serum suPAR (Supplementary Figure 1(A,B)). We defined that the effect of amiloride in decreasing uACR in DKD may be associated with the decline of urinary suPAR.

### The safety of amiloride during the treatment periods

Among ten DKD patients enrolled, one patient in the amiloride/HCTZ group and one patient in the HCTZ group experienced hyperkalemia during the treatment periods. Serum K^+^ in the amiloride/HCTZ group increased by 0.1 (−0.1, 0.9) mmol/L while 0.0 (−0.3, 0.2) mmol/L in the HCTZ group. Treatment effect on serum K^+^ in the amiloride/HCTZ group had no significant difference compared with those in the HCTZ group. Additionally, outcomes including changes in blood pressure, weight, serum Na^+^, serum uric acid, cholesterol, triglycerides before and after treatment were compared between the amiloride/HCTZ and HCTZ group. Analysis of variance for the two-period crossover design showed that there was no significant difference in side effects between groups (Table 3).

**Table 3.** Comparison of side effect between amiloride and HCTZ over the course of the study.

	Amiloride/HCTZ(*N* = 10)	HCTZ(*N* = 10)	F-value	P-value
Serum K^+^ (mmol/L)			2.348	0.164
Start of treatment	4.5 (4.1, 4.6)	4.7 (4.2, 4.9)		
End of treatment	4.7 (4.1, 5.4)	4.7 (4.1, 4.9)		
Changes of serum K^+^	0.1 (−0.1, 0.9)	0.0 (−0.3, 0.2)		
Serum Na^+^ (mmol/L)			2.308	0.167
Start of treatment	141.0 (138.9, 142.6)	141.5 (138.8, 142.5)		
End of treatment	141.8 (138.0, 142.9)	141.4 (141.0, 142.7)		
Changes of serum Na^+^	0.0 (−3.3, 2.5)	−0.5 (−1.5, 3.0)		
Serum Unic (umol/L)			0.058	0.816
Start of treatment	356.5 (289.0, 447.0)	380.5 (299.0, 419.8)		
End of treatment	380.5 (283.5, 407.0)	366.0 (283.3, 457.8)		
Changes of Serum Unic	1.0 (−70.0, 60.5)	10.5 (−79.3, 68.8)		
Cholesterol (mmol/L)			1.413	0.269
Start of treatment	5.4 (4.4, 6.0)	4.8 (4.7, 5.9)		
End of treatment	5.0 (3.9, 5.5)	4.8 (4.0, 6.2)		
Changes of cholesterol	−0.3 (−0.8, 0.1)	0.1 (−0.7, 0.4)		
Triglyceride (mmol/L)			1.212	0.303
Start of treatment	2.0 (1.1, 2.4)	1.6 (1.2, 2.6)		
End of treatment	1.7 (1.2, 2.5)	1.6 (1.0, 2.5)		
Changes of triglyceride	0.1 (−0.3, 0.3)	−0.3 (−0.5, −0.1)		
Fasting glucose (mmol/L)			0.961	0.356
Start of treatment	8.3 (6.2, 10.5)	7.8 (5.8, 11.0)		
End of treatment	7.1 (5.8, 8.2)	5.7 (4.8, 6.9)		
Changes of fasting glucose	−1.1 (−2.8, 0.2)	−1.3 (−4.5, −0.3)		
SBP (mmHg)			0.001	0.975
Start of treatment	135.0 (125.0, 137.0)	131.5 (123.5, 141.5)		
End of treatment	132.5 (125.3, 139.5)	130.5 (124.0, 141.5)		
Changes of SBP	1.0 (−5.5, 4.0)	0.0 (−5.0, 6.0)		
DBP (mmHg)			2.826	0.131
Start of treatment	74.5 (70.0, 76.5)	74.0 (71.5, 78.5)		
End of treatment	77.5 (72.8, 82.8)	75.0 (70.3, 79.3)		
Changes of DBP	3.0 (−2.0, 10.5)	−1.0 (−9.0, 5.3)		
Weight (kg)			3.404	0.102
Start of treatment	64.6 (57.8, 70.8)	64.2 (57.4, 70.9)		
End of treatment	64.2 (57.0, 70.5)	64.3 (57.1, 71.8)		
Changes of weight	−0.4 (0.9, 0.0)	0.3 (−0.4, 1.0)		

SBP: Systolic blood pressure; DBP: Diastolic blood pressure.

## Discussion

DKD is the most common cause of CKD and ESRD worldwide and develops in approximately 40% of type 2 diabetic patients [18]. DKD is a common microvascular complication of diabetes mellitus (DM) and most of the risk for all-cause and cardiovascular disease mortality in DM patients is related to the presence of DKD [19].

Progressively larger amounts of albuminuria are the most common manifestation in DKD and then classically followed by a relentless decline in kidney function, renal impairment, and ultimately ESRD. Albuminuria is a well-known factor that links podocyte damage to tubulointerstitial injury, which triggers tubulointerstitial inflammation and fibrogenesis, and accelerates the decline of renal function [20]. Therefore, reducing albuminuria in DKD is particularly important to delay its progression and improve outcomes.

The impairment of the glomerular filtration barrier is an important pathophysiological basis for albuminuria in DKD. The glomerular filtration barrier consists of endothelial cells, the glomerular basement membrane (GBM), and podocytes. Podocytes have a prominent role in maintaining the integrity of the glomerular filter. Podocytes injury would lead to the effacement of foot processes and the detachment or apoptosis of podocytes [21]. The loss of integrity of the podocyte seal results in the diffusion and convection of proteins from the circulation into the urinary space and finally leads to the occurrence of albuminuria [22]. Moreover, several pathogenic mechanisms involved in podocyte injury would beget ultrastructural changes in podocytes and albuminuria. Previous researches had found that mutation of the gene encoding the podocyte-expressed protein nephrin would result in congenital albuminuria [23]. Disorder of cross-talk between podocytes and the other cells involved in the glomerular filtration barrier might also lead to albuminuria. Several studies [19,24–26] had found that the podocyte lesion is the primary causes for glomerular diseases characterized by massive proteinuria, such as minimal change nephropathy (MCD), focal glomerulosclerosis (FSGS), membranous nephropathy (MN), lupus nephritis (LN), DKD and is related to disease progression.

Previous findings [11] had indicated that increasing expression of uPAR in podocytes would result in the podocytes lesion and the occurrence of albuminuria. uPAR is a multidomain glycoprotein tethered to the cell membrane with a glycosylphosphotidylinositol (GPI) anchor and was originally identified as a saturable binding site for urokinase on the cell surface. uPAR is required for activation of β3 integrin within the kidney podocyte, which is one of the main proteins that anchors podocytes to the underlying GBM. Increased β3 integrin activation within podocytes would cause accelerated podocyte foot process dynamics, which dysregulates the shape and function of the glomerular filtration barrier. Wei [11] et al. had found that lipopolysaccharide (LPS) injection would induce the higher expression of uPAR in podocytes. Mice lacking uPAR were protected from LPS-mediated proteinuria but develop the disease after expression of a constitutively active β3 integrin. Knockdown of uPAR in podocytes would decrease the number of migrating podocytes and promote motility, which showed a physiological role for uPAR signaling in the regulation of kidney permeability. Moreover, Zhao [27] et al. had demonstrated that urinary suPAR was specifically elevated in primary FSGS and was related to disease severity. The elevated urinary suPAR could activate β3 integrin on human podocytes. Therefore, it indicated that restraining the expression of uPAR in podocytes may be a potential therapeutic target for the clinical management of albuminuria in DKD.

Amiloride is a Na channel blocker and inhibits the Na^+^/H^+^ and Na^+^/Ca^2+^ antiporters, which have been widely used as a diuretic. Previous studies had suggested that amiloride could inhibit the expression of uPAR in tumor-infiltrating lymphocytes [28] and colon cancer cells [29,30]. Our former works had indicated that amiloride was able to inhibit the expression of uPAR and thereby reduced the mobility of podocytes, which provided a new therapeutic paradigm for the clinical management of albuminuria. Therefore, the effect of amiloride in decreasing albuminuria in DKD was explored in this prospective, crossover, open-label study.

In this crossover design, amiloride induced a significant decrease of uACR in DKD. Additionally, the decrease of serum and urinary suPAR in the amiloride/HCTZ group was significant compared with the HCTZ group. Correlation analysis had also shown that the levels of urinary suPAR were positively associated with uPCR and uACR. Based on the above results, we concluded that amiloride was able to reduce albuminuria in DKD and may be associated with the decreased levels of urinary suPAR, which was an indicator for disease severity [27]. Recent researches had revealed that urinary suPAR was significantly increased in patients with massive proteinuria and decreased accompanying with the remission of proteinuria. Mette Staehr [28] et al. had demonstrated that urinary uPA protein and activity were increased in rats treated with puromycin aminonucleoside. And amiloride could inhibit urinary uPA activity, which attenuated plasminogen activation and urine protease activity. Our studies had also found that amiloride could reduce urinary and serum suPAR, which may be a relevant target for amiloride in reducing albuminuria.

As Na channel blocker, amiloride would inhibit the exchange of Na^+^–K^+^ and Na^+^–H^+^ in distal tubules and collecting ducts of the kidney, thus increasing the excretion of Na^+^ and water and reducing the excretion of K^+^ and H^+^. Therefore, hyperkalemia and hyponatremia were the most common side effect of amiloride. In this study, significant hyperkalemia occurred in two patients and no patients experienced hyponatremia. And the serum potassium in both patients could return to normal after hypokalemia treatment. Additionally, there was no significant influence in fasting glucose, cholesterol, triglyceride, unic acid with amiloride/HCTZ when compared with HCTZ. Furthermore, amiloride would substantially reduce the SBP and volume status by blocking both ENaC and urokinase [31,32]. However, we had not found significant reductions in weight or SBP with amiloride/HCTZ compared with HCTZ. Our inability to detect significant effects on blood pressure and weight may be associated with the small sample size in this study. Unruh [33] et al. had also demonstrated the safety of amiloride in patients with type 2 diabetes, normal renal function, and proteinuria, which provided important research evidence for amiloride’s application in decreasing albuminuria in DKD.

The strength of this study mainly discussed the effect and safety of amiloride in decreasing albuminuria in DKD patients, which would provide a new application for the traditional diuretic. However, our study still has some limitations. First of all, we only enrolled small sizes sample from a single-center, and long-term renal prognosis had not been explored in DKD patients. Furthermore, the follow-up period should be extended in order to investigate the long-term effect of amiloride in DKD.

## Conclusions

Amiloride could induce a significant decrease of albuminuria, which was accompanied by the significant decline of urinary suPAR. No serious side effects were found in DKD patients using amiloride during treatment periods. In summary, amiloride might be an efficient and safe treatment strategy for decreasing albuminuria in DKD.

## Supplementary Material

Supplemental Material

## Data Availability

The datasets analyzed during the current study are available within the article and Supplementary file.

## Abbreviations

DKD: diabetic kidney diseases
uPCR: urine protein creatinine ratio
uACR: urinary albumin creatinine ratio
suPAR: soluble urokinase-type plasminogenactivator receptor
HCTZ: hydrochlorothiazide
