# Supplementary figures and images for: The effect of amiloride in decreasing albuminuria in patients with diabetic kidney diseases: a prospective, crossover, open-label study
Source: Ren Fail. 2021 Mar 3;43(1):452–9. doi: 10.1080/0886022X.2021.1892759 (PMC7935116; doi:10.1080/0886022X.2021.1892759)

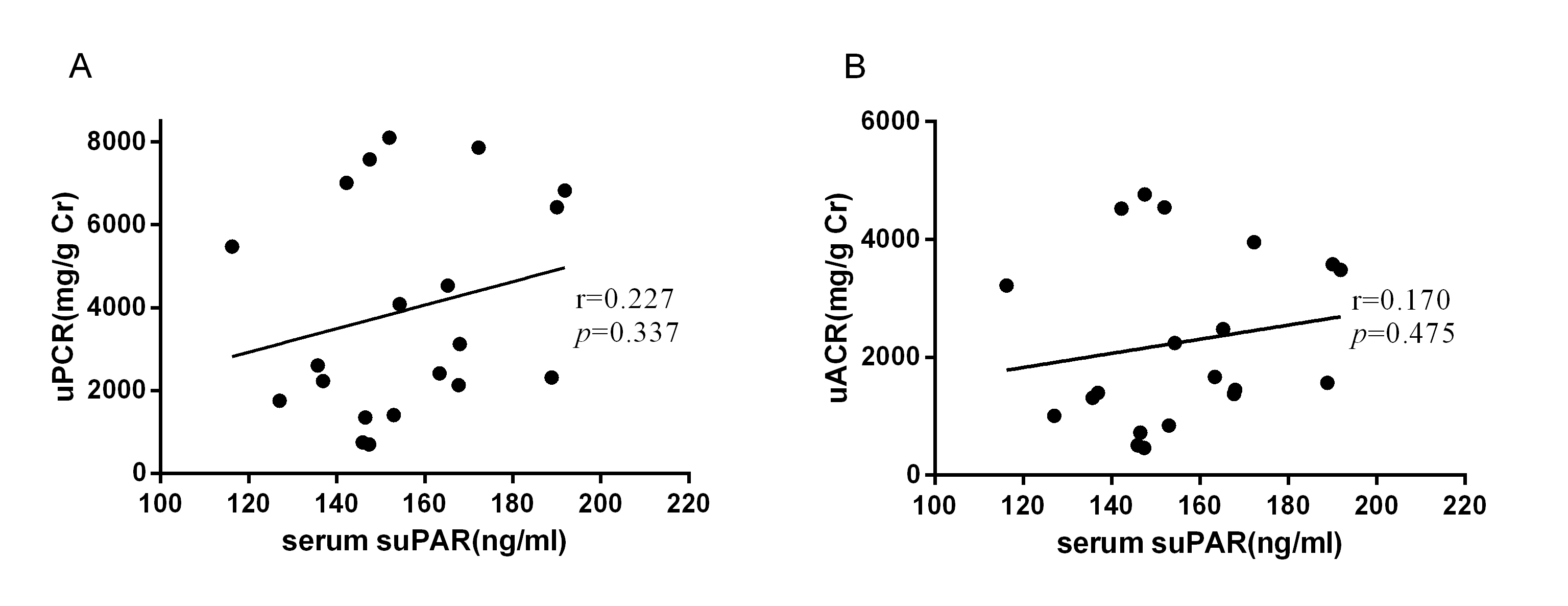

Supplement: Supplemental Material [file IRNF_A_1892759_SM2853.jpg]
